# Coexistence of Chronic Lymphocytic Leukemia and Acute Myeloid Leukemia

**DOI:** 10.4274/tjh.2016.0106

**Published:** 2016-12-01

**Authors:** Ivana Milosevic

**Affiliations:** 1 University of Novi Sad Faculty of Medicine, Clinical Center of Vojvodina, Novi Sad, Serbia

**Keywords:** Chronic lymphocytic leukemia, Acute myeloid leukemia, therapy

A 76-year-old man presented with leukocytosis (86x109/L), fever, pneumonia, and significant weight loss. He had a history of chronic lymphocytic leukemia diagnosed 5 years earlier and he responded with partial remission to treatment with continuous low doses of chlorambucil.

Analysis of the blood smear, bone marrow aspiration, and bone marrow biopsy revealed the predomination of small lymphocytes, but 22% of the cells were blasts negative with cytochemical staining (Figure 1). Flow cytometric analysis showed two distinct populations: 65% of cells were small to moderate in size and CD19+, CD45+, CD5+, and CD20+/-, while 30% of cells were large, CD34+, CD13+, HLA DR+, CD65+, CD45+, and MPO weakly positive and CD33, CD14, CD15, and CD16 negative. Immunophenotyping confirmed the coexistence of chronic lymphocytic leukemia and poorly differentiated acute myeloid leukemia. Conventional cytogenetic testing did not show any chromosomal abnormalities.

The patient was treated with intensive antibiotherapy and received one course of chemotherapy, but he did not achieve remission and died 2 months later.

The coexistence of chronic lymphocytic leukemia and acute myeloid leukemia is rare [1]. Therapy-related acute myeloid leukemia can develop after treatment of chronic lymphocytic leukemia with alkylating agents, nucleoside analogs, or combination chemotherapy, but the two leukemias can also originate independently [2,3].

Conflict of Interest: The author of this paper has no conflicts of interest, including specific financial interests, relationships, and/or affiliations relevant to the subject matter or materials included.

## Figures and Tables

**Figure 1:**
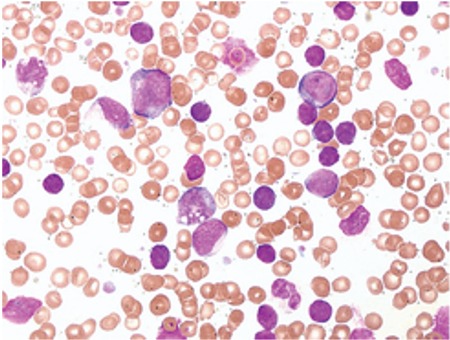
Chronic lymphocytic leukemia cells and acute myeloid leukemia cells in the peripheral blood smear.
